# Redetermination of 3-methyl­benzoic acid

**DOI:** 10.1107/S1600536811003849

**Published:** 2011-02-05

**Authors:** Rodolfo Moreno-Fuquen, Regina De Almeida Santos, Alan R. Kennedy

**Affiliations:** a Departamento de Química - Facultad de Ciencias, Universidad del Valle, Apartado 25360, Santiago de Cali, Colombia; b Instituto de Química, IQSC, Universidade de São Paulo, São Carlos, Brazil; c WestCHEM, Department of Pure and Applied Chemistry, University of Strathclyde, 295 Cathedral Street, Glasgow G1 1XL, Scotland

## Abstract

The asymmetric unit of the title compound, C_8_H_8_O_2_, contains two crystallographically independent mol­ecules, which form dimers linked by O⋯H—O hydrogen bonds. The benzene rings in the dimers are inclined at a dihedral angle of 7.30 (8)° and both methyl groups display rotational disorder. This redetermination results in a crystal structure with significantly higher precision than the original determination [Ellas & García-Blanco (1963 ▶). *Acta Cryst*. **16**, 434], in which the authors reported only the unit-cell parameters and space group, without any detailed information on the atomic arrangement. In the crystal, dimers are connected by weak C—H⋯O inter­actions, forming *R*_2_^2^(10) and *R*_4_^4^(18) rings along [110] and an infinite zigzag chain of dimers along the [001] direction also occurs.

## Related literature

For a report of the unit-cell dimensions and space group of the title compound, see: Ellas & García-Blanco (1963 ▶). For comparisons with other hydrogen-bond donors, see: Moreno-Fuquen *et al.* (1997 ▶, 2009 ▶, 2011 ▶). For related structures, see: Barcon *et al.* (1997 ▶). For bond-length data, see: Allen *et al.* (1987 ▶). For a structural discussion of hydrogen bonding, see: Desiraju & Steiner (1999 ▶). For general analysis of inter­molecular inter­actions, see: Nardelli (1995 ▶) and for graph-set notation of hydrogen-bond patterns, see: Etter (1990 ▶).
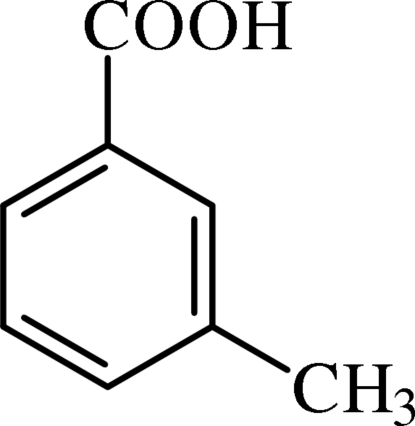


## Experimental

### 

#### Crystal data

C_8_H_8_O_2_
*M* *_r_* = 136.14
Monoclinic, 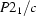

*a* = 10.3693 (9) Å
*b* = 8.1844 (7) Å
*c* = 16.4715 (17) Å
β = 92.836 (9)°
*V* = 1396.2 (2) Å^3^
*Z* = 8
Mo *K*α radiation
μ = 0.09 mm^−1^
*T* = 123 K
0.22 × 0.18 × 0.06 mm

#### Data collection

Oxford Diffraction Gemini S diffractometer
13219 measured reflections
3705 independent reflections
1898 reflections with *I* > 2σ(*I*)
*R*_int_ = 0.055

#### Refinement

*R*[*F* ^2^ > 2σ(*F* ^2^)] = 0.045
*wR*(*F* ^2^) = 0.104
*S* = 0.83
3705 reflections
187 parameters
H atoms treated by a mixture of independent and constrained refinement
Δρ_max_ = 0.23 e Å^−3^
Δρ_min_ = −0.26 e Å^−3^

### 

Data collection: *CrysAlis RED* (Oxford Diffraction, 2009 ▶); cell refinement: *CrysAlis RED*; data reduction: *CrysAlis CCD* (Oxford Diffraction, 2009 ▶); program(s) used to solve structure: *SHELXS97* (Sheldrick, 2008 ▶); program(s) used to refine structure: *SHELXL97* (Sheldrick, 2008 ▶); molecular graphics: *ORTEP-3 for Windows* (Farrugia, 1997 ▶) and *Mercury* (Macrae *et al.*, 2006 ▶); software used to prepare material for publication: *SHELXL97*.

## Supplementary Material

Crystal structure: contains datablocks I, global. DOI: 10.1107/S1600536811003849/sj5096sup1.cif

Structure factors: contains datablocks I. DOI: 10.1107/S1600536811003849/sj5096Isup2.hkl

Additional supplementary materials:  crystallographic information; 3D view; checkCIF report

## Figures and Tables

**Table 1:** Hydrogen-bond geometry (Å, °)

*D*—H⋯*A*	*D*—H	H⋯*A*	*D*⋯*A*	*D*—H⋯*A*
O1—H1*H*⋯O4	0.984 (18)	1.634 (19)	2.6149 (16)	173.7 (16)
O3—H2*H*⋯O2	0.998 (19)	1.623 (19)	2.6205 (16)	177.6 (16)
C7—H7⋯O1^i^	0.95	2.70	3.4520 (18)	137
C16—H16*E*⋯O1^ii^	0.98	2.54	3.448 (2)	154
C14—H14⋯O2^iii^	0.95	2.67	3.460 (2)	141

Symmetry codes: (i) 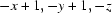; (ii) 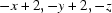; (iii) 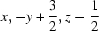.

**Fig. 1.**
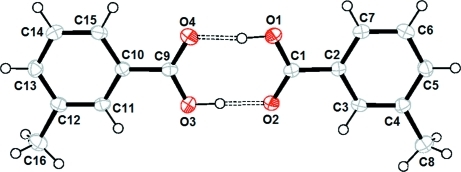
The asymmetric unit of the title compound with the atomic labelling scheme and displacement ellipsoids drawn at the 50% probability level. H bonds are drawn as open dashed lines and only one component of the disordered methyl groups is shown.

**Fig. 2.**
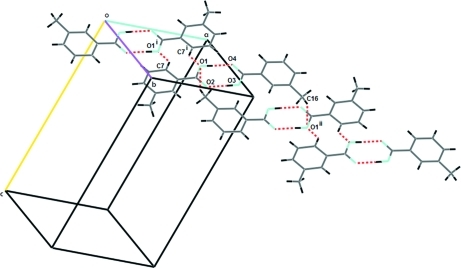
Part of the crystal structure of (I), showing the formation of R22(10) and R44(18) rings, running along [110]. H bonds are drawn as dashed lines. Symmetry codes: (i) -x + 1, -y + 1,-z; (ii) -x + 2,-y + 2,-z.

**Fig. 3.**
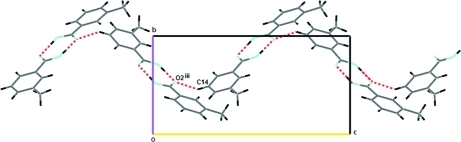
Part of the crystal structure of (I), showing the formation of an infinite zigzag chain of dimers along the [001] direction. H bonds are drawn as dashed lines. Symmetry code: (iii) x,-y + 1/2 + 1,+z - 1/2.

## supplementary crystallographic information

### Comment

The title compound (I) was investigated in a continuation of our studies on the formation of molecular complexes from N-oxide derivatives with different hydrogen bond donors (Moreno-Fuquen *et al.*, 1997, 2009, 2011). The structure of a similar molecule was taken to compare with the title molecule (Barcon *et al.*, 1997). A perspective view of the dimeric hydrogen-bonding in (I), showing the  atomic numbering scheme, is given in Figure 1. Both molecules of *m*-toluic acid are held together by intermolecular O···H—O hydrogen bonds of moderate character (Desiraju & Steiner, 1999). Indeed, carbonylic O4 and O2 atoms are linked to O1 and O3 atoms with O···O distances of 2.6149 (16) and 2.6205 (16)Å respectively. The two aromatic rings in (I) form a dihedral angle of 7.30 (8)°. Both methyl groups display rotational disorder. The rotamer ratio found in this modeling was 50:50. In (I), Fig. 1, bond lengths and bond angles are in normal ranges (Allen *et al.*, 1987). The title crystal structure also exhibits other weak C—H···O interactions (see Table 1, Nardelli, 1995). Indeed, in a first substructure, atom C7 acts as hydrogen bond donor to O1^i^ in the molecule at (-*x* + 1,-*y* + 1,-*z*) and the C16 atom acts as hydrogen bond donor to O1^ii^ in the molecule at (-*x* + 2,-*y* + 2,-*z*). The propagation of these interactions generates rings with graph-set notation *R*_2_^2^(10) and *R*_4_^4^(18) (Etter, 1990), running along the [110] direction (see Fig. 2). In a second substructure, atom C14 acts as a hydrogen bond donor to O2^iii^ in the molecule at (*x*,-*y* + 1/2 + 1,+*z* - 1/2). The propagation of this interaction forms an infinite zigzag chain of dimers along the [001] direction (see Fig. 3). All of these interactions in the [110] and [001] directions define the bulk structure of the crystal.

### Experimental

3-Methylbenzoic acid (0.545 g, 4 mmol) (Aldrich) was disssolved in ethanol (200 ml). The solution was left to evaporate slowly at room temperature. After three days, colourless crystals of a good quality suitable for X-ray analysis were obtained. *M*. p. 383 (1) K

### Refinement

All non-hydrogen atoms were identified by direct methods. All H atoms were observed in a difference Fourier map. The H-atoms in (I) were placed geometrically [C—H= 0.95 Å for aromatic, C—H= 0.98 Å for methyl, *U*_iso_(H) (1.2 and 1.5 times *U*_eq_ of the parent atom respectivelly]. The coordinates of the H1H and H2H hydroxyl H atoms were refined.

### Crystal data

**Table:**

C_8_H_8_O_2_	*F*(000) = 576
*M**_r_* = 136.14	*D*_x_ = 1.295 Mg m^−^^3^
Monoclinic, *P*2_1_/*c*	Melting point: 383(1) K
Hall symbol: -P 2ybc	Mo *K*α radiation, λ = 0.71073 Å
*a* = 10.3693 (9) Å	Cell parameters from 2724 reflections
*b* = 8.1844 (7) Å	θ = 2.5–31.0°
*c* = 16.4715 (17) Å	µ = 0.09 mm^−^^1^
β = 92.836 (9)°	*T* = 123 K
*V* = 1396.2 (2) Å^3^	Tablet, colourless
*Z* = 8	0.22 × 0.18 × 0.06 mm

### Data collection

**Table:**

Oxford Diffraction Gemini S diffractometer	1898 reflections with *I* > 2σ(*I*)
Radiation source: fine-focus sealed tube	*R*_int_ = 0.055
graphite	θ_max_ = 29.0°, θ_min_ = 2.8°
ω scans	*h* = −12→14
13219 measured reflections	*k* = −11→11
3705 independent reflections	*l* = −22→21

### Refinement

**Table:**

Refinement on *F*^2^	Primary atom site location: structure-invariant direct methods
Least-squares matrix: full	Secondary atom site location: difference Fourier map
*R*[*F*^2^ > 2σ(*F*^2^)] = 0.045	Hydrogen site location: inferred from neighbouring sites
*wR*(*F*^2^) = 0.104	H atoms treated by a mixture of independent and constrained refinement
*S* = 0.83	*w* = 1/[σ^2^(*F*_o_^2^) + (0.0487*P*)^2^] where *P* = (*F*_o_^2^ + 2*F*_c_^2^)/3
3705 reflections	(Δ/σ)_max_ < 0.001
187 parameters	Δρ_max_ = 0.23 e Å^−^^3^
0 restraints	Δρ_min_ = −0.26 e Å^−^^3^

### Special details

**Table:**

Geometry. All e.s.d.'s (except the e.s.d. in the dihedral angle between two l.s. planes) are estimated using the full covariance matrix. The cell e.s.d.'s are taken into account individually in the estimation of e.s.d.'s in distances, angles and torsion angles; correlations between e.s.d.'s in cell parameters are only used when they are defined by crystal symmetry. An approximate (isotropic) treatment of cell e.s.d.'s is used for estimating e.s.d.'s involving l.s. planes.
Refinement. Refinement of *F*^2^ against ALL reflections. The weighted *R*-factor *wR* and goodness of fit *S* are based on *F*^2^, conventional *R*-factors *R* are based on *F*, with *F* set to zero for negative *F*^2^. The threshold expression of *F*^2^ > σ(*F*^2^) is used only for calculating *R*-factors(gt) *etc*. and is not relevant to the choice of reflections for refinement. *R*-factors based on *F*^2^ are statistically about twice as large as those based on *F*, and *R*- factors based on ALL data will be even larger.

### Fractional atomic coordinates and isotropic or equivalent isotropic displacement parameters (Å^2^)

**Table:**

	*x*	*y*	*z*	*U*_iso_*/*U*_eq_	Occ. (<1)
O1	0.71126 (10)	0.51712 (14)	0.01320 (7)	0.0292 (3)	
H1H	0.7709 (16)	0.584 (2)	−0.0173 (11)	0.044*	
O2	0.86464 (10)	0.53282 (13)	0.11340 (7)	0.0282 (3)	
O3	1.01280 (11)	0.71203 (14)	0.02472 (7)	0.0305 (3)	
H2H	0.9547 (16)	0.646 (2)	0.0585 (11)	0.046*	
O4	0.85611 (10)	0.70355 (14)	−0.07354 (7)	0.0331 (3)	
C1	0.75522 (14)	0.48677 (17)	0.08633 (9)	0.0210 (3)	
C2	0.66970 (14)	0.39351 (17)	0.13857 (9)	0.0206 (3)	
C3	0.71441 (14)	0.34920 (18)	0.21672 (9)	0.0224 (4)	
H3	0.7986	0.3812	0.2357	0.027*	
C4	0.63808 (15)	0.25912 (19)	0.26726 (10)	0.0261 (4)	
C5	0.51487 (15)	0.2154 (2)	0.23776 (10)	0.0285 (4)	
H5	0.4613	0.1529	0.2711	0.034*	
C6	0.46861 (15)	0.2611 (2)	0.16069 (10)	0.0290 (4)	
H6	0.3836	0.2314	0.1422	0.035*	
C7	0.54596 (14)	0.35009 (18)	0.11062 (10)	0.0247 (4)	
H7	0.5146	0.3811	0.0577	0.030*	
C8	0.68850 (17)	0.2078 (2)	0.35071 (10)	0.0404 (5)	
H8A	0.6218	0.1456	0.3774	0.061*	0.50
H8B	0.7111	0.3050	0.3831	0.061*	0.50
H8C	0.7653	0.1394	0.3460	0.061*	0.50
H8D	0.7770	0.2478	0.3602	0.061*	0.50
H8E	0.6877	0.0883	0.3545	0.061*	0.50
H8F	0.6335	0.2539	0.3917	0.061*	0.50
C9	0.96396 (14)	0.75391 (18)	−0.04584 (10)	0.0229 (4)	
C10	1.04204 (14)	0.86537 (18)	−0.09412 (10)	0.0217 (3)	
C11	1.15930 (14)	0.92586 (18)	−0.06163 (10)	0.0236 (4)	
H11	1.1880	0.8960	−0.0080	0.028*	
C12	1.23462 (14)	1.02899 (18)	−0.10643 (10)	0.0244 (4)	
C13	1.19025 (15)	1.07092 (19)	−0.18470 (10)	0.0284 (4)	
H13	1.2408	1.1415	−0.2161	0.034*	
C14	1.07403 (15)	1.01207 (19)	−0.21794 (10)	0.0287 (4)	
H14	1.0454	1.0425	−0.2715	0.034*	
C15	0.99964 (14)	0.90856 (19)	−0.17276 (10)	0.0256 (4)	
H15	0.9201	0.8673	−0.1954	0.031*	
C16	1.36251 (15)	1.0906 (2)	−0.07154 (11)	0.0331 (4)	
H16A	1.4020	1.1616	−0.1112	0.050*	0.50
H16B	1.4197	0.9977	−0.0589	0.050*	0.50
H16C	1.3491	1.1525	−0.0217	0.050*	0.50
H16D	1.3785	1.0462	−0.0167	0.050*	0.50
H16E	1.3608	1.2102	−0.0690	0.050*	0.50
H16F	1.4315	1.0554	−0.1062	0.050*	0.50

### Atomic displacement parameters (Å^2^)

**Table:**

	*U*^11^	*U*^22^	*U*^33^	*U*^12^	*U*^13^	*U*^23^
O1	0.0287 (6)	0.0374 (7)	0.0211 (6)	−0.0020 (5)	−0.0018 (5)	0.0040 (6)
O2	0.0249 (6)	0.0303 (6)	0.0290 (7)	−0.0045 (5)	−0.0046 (5)	0.0047 (5)
O3	0.0291 (6)	0.0339 (6)	0.0284 (7)	−0.0014 (5)	0.0009 (5)	0.0092 (6)
O4	0.0247 (6)	0.0380 (7)	0.0361 (7)	−0.0051 (5)	−0.0029 (5)	0.0029 (6)
C1	0.0227 (8)	0.0188 (7)	0.0215 (9)	0.0042 (7)	0.0000 (7)	−0.0023 (7)
C2	0.0220 (8)	0.0183 (7)	0.0217 (9)	0.0024 (6)	0.0012 (7)	−0.0023 (7)
C3	0.0185 (8)	0.0257 (8)	0.0227 (9)	0.0011 (6)	−0.0023 (7)	−0.0022 (7)
C4	0.0267 (8)	0.0276 (9)	0.0240 (9)	0.0012 (7)	0.0004 (7)	0.0004 (7)
C5	0.0257 (9)	0.0313 (9)	0.0287 (10)	−0.0030 (7)	0.0042 (8)	0.0013 (8)
C6	0.0221 (8)	0.0331 (9)	0.0314 (10)	−0.0015 (7)	−0.0032 (7)	−0.0025 (8)
C7	0.0247 (8)	0.0262 (8)	0.0228 (9)	0.0023 (7)	−0.0030 (7)	−0.0006 (7)
C8	0.0397 (11)	0.0515 (12)	0.0292 (10)	−0.0039 (9)	−0.0047 (9)	0.0111 (9)
C9	0.0223 (8)	0.0219 (8)	0.0242 (9)	0.0032 (7)	−0.0020 (7)	−0.0007 (7)
C10	0.0212 (8)	0.0188 (7)	0.0251 (9)	0.0033 (6)	0.0015 (7)	0.0002 (7)
C11	0.0242 (8)	0.0231 (8)	0.0233 (9)	0.0057 (7)	−0.0015 (7)	−0.0004 (7)
C12	0.0227 (8)	0.0221 (8)	0.0283 (9)	0.0034 (7)	0.0005 (7)	−0.0029 (7)
C13	0.0285 (9)	0.0240 (8)	0.0331 (10)	−0.0019 (7)	0.0038 (8)	0.0044 (8)
C14	0.0301 (9)	0.0295 (9)	0.0261 (9)	0.0030 (7)	−0.0025 (8)	0.0049 (8)
C15	0.0216 (8)	0.0246 (8)	0.0300 (10)	0.0005 (7)	−0.0040 (7)	0.0008 (7)
C16	0.0280 (9)	0.0316 (9)	0.0396 (11)	−0.0023 (8)	−0.0012 (8)	−0.0030 (8)

### Geometric parameters (Å, °)

**Table:**

O1—C1	1.2909 (17)	C8—H8D	0.9800
O1—H1H	0.984 (18)	C8—H8E	0.9800
O2—C1	1.2562 (17)	C8—H8F	0.9800
O3—C9	1.2911 (18)	C9—C10	1.478 (2)
O3—H2H	0.998 (19)	C10—C15	1.393 (2)
O4—C9	1.2563 (16)	C10—C11	1.395 (2)
C1—C2	1.478 (2)	C11—C12	1.387 (2)
C2—C7	1.3878 (19)	C11—H11	0.9500
C2—C3	1.394 (2)	C12—C13	1.390 (2)
C3—C4	1.389 (2)	C12—C16	1.506 (2)
C3—H3	0.9500	C13—C14	1.385 (2)
C4—C5	1.391 (2)	C13—H13	0.9500
C4—C8	1.506 (2)	C14—C15	1.387 (2)
C5—C6	1.386 (2)	C14—H14	0.9500
C5—H5	0.9500	C15—H15	0.9500
C6—C7	1.385 (2)	C16—H16A	0.9800
C6—H6	0.9500	C16—H16B	0.9800
C7—H7	0.9500	C16—H16C	0.9800
C8—H8A	0.9800	C16—H16D	0.9800
C8—H8B	0.9800	C16—H16E	0.9800
C8—H8C	0.9800	C16—H16F	0.9800
			
C1—O1—H1H	112.5 (10)	H8E—C8—H8F	109.5
C9—O3—H2H	115.1 (10)	O4—C9—O3	122.84 (15)
O2—C1—O1	122.99 (14)	O4—C9—C10	120.58 (14)
O2—C1—C2	120.38 (13)	O3—C9—C10	116.58 (13)
O1—C1—C2	116.63 (13)	C15—C10—C11	119.73 (14)
C7—C2—C3	119.96 (14)	C15—C10—C9	120.06 (14)
C7—C2—C1	120.70 (13)	C11—C10—C9	120.20 (14)
C3—C2—C1	119.34 (13)	C12—C11—C10	121.00 (14)
C4—C3—C2	121.21 (14)	C12—C11—H11	119.5
C4—C3—H3	119.4	C10—C11—H11	119.5
C2—C3—H3	119.4	C11—C12—C13	118.31 (14)
C3—C4—C5	117.90 (14)	C11—C12—C16	120.61 (14)
C3—C4—C8	120.86 (14)	C13—C12—C16	121.07 (15)
C5—C4—C8	121.23 (15)	C14—C13—C12	121.48 (15)
C6—C5—C4	121.38 (16)	C14—C13—H13	119.3
C6—C5—H5	119.3	C12—C13—H13	119.3
C4—C5—H5	119.3	C13—C14—C15	119.76 (15)
C7—C6—C5	120.18 (15)	C13—C14—H14	120.1
C7—C6—H6	119.9	C15—C14—H14	120.1
C5—C6—H6	119.9	C14—C15—C10	119.71 (14)
C6—C7—C2	119.35 (14)	C14—C15—H15	120.1
C6—C7—H7	120.3	C10—C15—H15	120.1
C2—C7—H7	120.3	C12—C16—H16A	109.5
C4—C8—H8A	109.5	C12—C16—H16B	109.5
C4—C8—H8B	109.5	H16A—C16—H16B	109.5
H8A—C8—H8B	109.5	C12—C16—H16C	109.5
C4—C8—H8C	109.5	H16A—C16—H16C	109.5
H8A—C8—H8C	109.5	H16B—C16—H16C	109.5
H8B—C8—H8C	109.5	C12—C16—H16D	109.5
C4—C8—H8D	109.5	H16A—C16—H16D	141.1
H8A—C8—H8D	141.1	H16B—C16—H16D	56.3
H8B—C8—H8D	56.3	H16C—C16—H16D	56.3
H8C—C8—H8D	56.3	C12—C16—H16E	109.5
C4—C8—H8E	109.5	H16A—C16—H16E	56.3
H8A—C8—H8E	56.3	H16B—C16—H16E	141.1
H8B—C8—H8E	141.1	H16C—C16—H16E	56.3
H8C—C8—H8E	56.3	H16D—C16—H16E	109.5
H8D—C8—H8E	109.5	C12—C16—H16F	109.5
C4—C8—H8F	109.5	H16A—C16—H16F	56.3
H8A—C8—H8F	56.3	H16B—C16—H16F	56.3
H8B—C8—H8F	56.3	H16C—C16—H16F	141.1
H8C—C8—H8F	141.1	H16D—C16—H16F	109.5
H8D—C8—H8F	109.5	H16E—C16—H16F	109.5
			
O2—C1—C2—C7	176.39 (14)	O4—C9—C10—C15	−3.5 (2)
O1—C1—C2—C7	−3.5 (2)	O3—C9—C10—C15	176.20 (14)
O2—C1—C2—C3	−3.6 (2)	O4—C9—C10—C11	177.45 (14)
O1—C1—C2—C3	176.54 (13)	O3—C9—C10—C11	−2.9 (2)
C7—C2—C3—C4	1.2 (2)	C15—C10—C11—C12	0.1 (2)
C1—C2—C3—C4	−178.85 (14)	C9—C10—C11—C12	179.20 (14)
C2—C3—C4—C5	−0.5 (2)	C10—C11—C12—C13	0.1 (2)
C2—C3—C4—C8	178.29 (15)	C10—C11—C12—C16	−178.59 (14)
C3—C4—C5—C6	−0.6 (2)	C11—C12—C13—C14	0.0 (2)
C8—C4—C5—C6	−179.39 (16)	C16—C12—C13—C14	178.60 (15)
C4—C5—C6—C7	1.0 (2)	C12—C13—C14—C15	−0.2 (2)
C5—C6—C7—C2	−0.3 (2)	C13—C14—C15—C10	0.4 (2)
C3—C2—C7—C6	−0.8 (2)	C11—C10—C15—C14	−0.4 (2)
C1—C2—C7—C6	179.30 (14)	C9—C10—C15—C14	−179.42 (14)

### Hydrogen-bond geometry (Å, °)

**Table:**

*D*—H···*A*	*D*—H	H···*A*	*D*···*A*	*D*—H···*A*
O1—H1H···O4	0.984 (18)	1.634 (19)	2.6149 (16)	173.7 (16)
O3—H2H···O2	0.998 (19)	1.623 (19)	2.6205 (16)	177.6 (16)
C7—H7···O1^i^	0.95	2.70	3.4520 (18)	137
C16—H16E···O1^ii^	0.98	2.54	3.448 (2)	154
C14—H14···O2^iii^	0.95	2.67	3.460 (2)	141

Symmetry codes:  (i) −*x*+1, −*y*+1, −*z*; (ii) −*x*+2, −*y*+2, −*z*; (iii) *x*, −*y*+3/2, *z*−1/2.
